# The protective effect of hydroalcoholic extract of Ginger (*Zingiber officinale* Rosc.) against iron-induced functional and histological damages in rat liver and kidney

**Published:** 2017

**Authors:** Firouzeh Gholampour, Fatemeh Behzadi Ghiasabadi, Seyed Mohammad Owji, Jaafar Vatanparast

**Affiliations:** 1 *Department of Biology, School of Sciences, Shiraz University, Shiraz, Iran*; 2 *Department of Pathology, School of Medicine, Shiraz University of Medical Sciences, Shiraz, Iran*

**Keywords:** Albumin, Ferrous Sulfate, Kidney, Lipid peroxidation, Liver, Zingiber officinale

## Abstract

**Objective:**

Iron overload in the body is related with toxic effects and threatens the health. The aim of this study was to evaluate the protective role of hydroalcoholic extract of ginger (*Zingiber officinale*) against ferrous sulfate-induced hepatic and renal functional disorders and histological damages in rats.

**Materials and Methods:**

The rats were divided into four groups (n=7): Sham, Sham + G.E (ginger extract, 400 mg/kg/day for 14 days), FS (ferrous sulfate, 30 mg/kg/day for 14 days), FS+G.E (ferrous sulfate, 30 mg/kg/day for 14 days; ginger extract, 400 mg/kg/day for 11 days from the fourth day of ferrous sulfate injection). After 24 hr, blood, urine and tissue samples were collected.

**Results:**

Compared with Sham and Sham + G.E groups, administration of ferrous sulfate resulted in liver and kidney dysfunction as evidenced by significantly higher levels of serum hepatic markers and bilirubin, and lower levels of serum albumin, total protein, triglyceride, cholesterol and glucose, as well as lower creatinine clearance and higher fractional excretion of sodium (p<0.001). This was accompanied by increased malondialdehyde levels and histological damages (p<0.001). In the FS + G.E, ginger extract significantly (p<0.01) reversed the levels of serum hepatic markers, renal functional markers and lipid peroxidation marker. Furthermore, it restored the levels of serum total protein, albumin, glucose, triglycerides and cholesterol and decreased bilirubin concentration in the blood. All these changes were corroborated by histological observations of liver and kidney.

**Conclusion:**

In conclusion, ginger extract appears to exert protective effects against ferrous sulfate-induced hepatic and renal toxicity by reducing lipid peroxidation and chelating iron.

## Introduction

Iron is one of the microminerals that is categorized as an essential nutrient (Pari et al. 2015 ▶). It is found in ferrous and ferric forms that can cycle reversibly between their oxidation states (Halliwell and Gutteridge 1990 ▶). Iron overload increases the formation of reactive oxygen species (ROS) which can damage the cell (Pari et al. 2015 ▶). It is considered that the interaction between iron and superoxide (O_2_^-^**)** generated via Fenton/Haber–Weiss reaction, leads to ROS formation (Kehrer 2000 ▶). When excessive amounts of ROS is produced, homeostasis will be disturbed and oxidative stress occurs.

Iron overdose causes accumulation of iron in the body. Liver is commonly affected by iron overload because it is the most active site of iron storage in the body (Papanastasiou et al. 2000 ▶). Besides, iron overload can lead to intra-lysosomal storage of iron in the kidney (Dimitriou et al. 2000 ▶) and causes renal tubular injury due to formation of hydroxyl (^•^OH) radical (Madhusudhan and Oberoi 2011 ▶). Zager et al. have shown that high concentrations of Fe^2+^ cause acute cytotoxicity in rat renal proximal tubular segments (Zager et al. 2004 ▶).

Medicinal plants with antioxidant potential are effective against various human diseases. *Zingiber officinale *Roscoe, commonly known as ginger, belongs *to Zingiberaceae* family. It is one of the oldest and intensively-researched medicinal plants which is commonly used in Iran and other countries of the world (Pari et al. 2015 ▶). Clinical and experimental data showed the anti-inflammatory (Thomson et al. 2002 ▶), antihypercholesterolemic (Bhandari et al. 2005 ▶), antihyperlipidaemic (Kadnur and Goyal 2005 ▶), antiemetic (Vishwakarma et al. 2002 ▶) and antitoxic (Egwurugwu et al. 2007 ▶) properties of *Z. officinale*. However, the protective role of hydroalcoholic extract of ginger against ferrous sulfate-induced liver and kidney injury has not been investigated. Hence, we investigated whether administration of hydroalcoholic extract of ginger offers protection against ferrous sulfate –induced functional disorders and tissue damages of the liver and kidney in rats.

## Materials and Methods

**Preparation of the extract**

**Plant material**

Purchased rhizomes of ginger were cultivated in a suitable environment. A voucher specimen was deposited at the Herbarium of Shiraz University (voucher No. 25052). The material was dried in the dark at room temperature before extraction.

**Extraction of the plant material**

Here, 100 g of finely-powdered dried ginger was submitted to extraction using 500 ml of 70% methanol and water at a ratio of 1:1 in a percolator apparatus for 72 hr. After extraction, the solvent was filtered and then evaporated in a rotary evaporator at 40 ºC. The dried extract weighed 30.5 g indicating a 30.5% yield. 

**Experimental procedure**

Male Wistar rats (250–300 g) were obtained from Razi institute, Shiraz, Iran. The animals were grouped (n=7), housed in polyacrylic cages and maintained under standard laboratory conditions (at 25±2 °C with 12 hr:12 h light/dark cycles). They had access to a standard pellet diet and water, *ad libitum*. The Ethics Committee of Shiraz University approved the study. The rats were divided into four groups: Sham (n=7), Sham+G.E (ginger extract 400 mg/kg/day dissolved in 1 ml distilled water and given by gavage for 14 days), FS (ferrous sulfate 30 mg/kg/day dissolved in 1 ml distilled water and given intraperitoneally (i.p.) for 14 days), FS+G.E (ferrous sulfate 30 mg/kg/day dissolved in 1 ml distilled water and given i.p. for 14 days; ginger extract 400 mg/kg/day dissolved in 1 ml distilled water and given by gavage for 11 days from the fourth day of ferrous sulfate injection). After 14 days of ferrous sulfate/ginger extract treatment, rats were placed in metabolic cages and urine was collected over a period of 24 hr. Thereafter, rats were anesthetized and blood samples were obtained from heart ventricles. Then, the liver and left kidney were quickly isolated. Parts of liver and kidney preserved for future histological examination and the rest was immediately snap-frozen in liquid nitrogen and stored at -70°C until further use. Rats were killed by injecting an overdose of anaesthetics.

**Renal functional assessments**

Urine samples were collected at the end of the 24-hr period and total volume was recorded. Urinary creatinine was measured by colorimetric methods (Prestige, Biolis24I, Japan) and used in conjunction with serum creatinine concentration and urine flow to calculate creatinine clearance (C_Cr_) using the standard formula (C_Cr _= [urine flow rate × urine concentration of creatinine]/[plasma concentration of creatinine]). Creatinine clearance was used as an indicator of glomerular function. Urinary Na^+^ was measured at the end of the 24-hr period and used in conjunction with serum Na^+^ to estimate the fractional excretion of Na^+^ (FE_Na_) using the standard formula (FE_Na_=[Absolute excretion of Na×100]/[Plasma concentration of Na×C_Cr_]).The FE_Na_ was used as an indicator of tubular dysfunction.

**Biochemical assays**

Alanine aminotransferase (ALT), aspartate aminotransferase (AST), lactate dehydrogenase (LDH) and alkaline phosphatase (ALP) activities in serum and liver tissue samples were measured by commercially available kits. Also, total protein, albumin, glucose, triglycerides and cholesterol concentrations in serum were measured by commercially available kits.

**Lipid peroxidation assay**

Malondialdehyde (MDA) levels were determined in tissue samples according to the methods of Heath & Packer (Heath and Packer, 1986). Malondialdehyde reacts with thiobarbituric acid (TBA) to produce a pink pigment that has a maximum absorption at 532 nm.

**Histopathological examinations**

Liver and kidney samples were fixed in buffered 10 % formalin. After dehydration through graded alcohol series, the samples were cleared in xylol. Then, liver and kidney samples were embedded in paraffin and 5-μm sections were obtained using a microtome (Erma, Japan). Routine staining with Prussian blue as well as hematoxylin and eosin, was done for each liver and kidney section. In a blinded fashion, each section was examined in at least 10 randomly selected non-overlapping fields under light microscope. In each liver section, we examined the degree of the presence of congestion and cellular degenerative changes. The renal histopathology was quantified for tubular necrosis, loss of brush borders, and formation of casts as well as vascular congestion. The level of each pathological manifestation was graded according to the observed changes as follows: none (0), less than 20 % (1), 21–40 % (2), 41-60% (3), 61–80 % (4), and greater than 80 % (5). The sum of all numerical scores in each group was taken as the total histopathological score.

**Statistical analysis**

Data are presented as mean ± SEM. They were assessed by one-way analysis of variance followed by Duncan’s *post-hoc* for comparison among groups. All data analyses were performed using SPSS version 22 software (SPSS Software, Chicago, IL, USA) and significance was considered at p≤0.05.

## Results

**Ginger extract and ferrous sulfate-induced changes in body weight**

**Figure 1:**
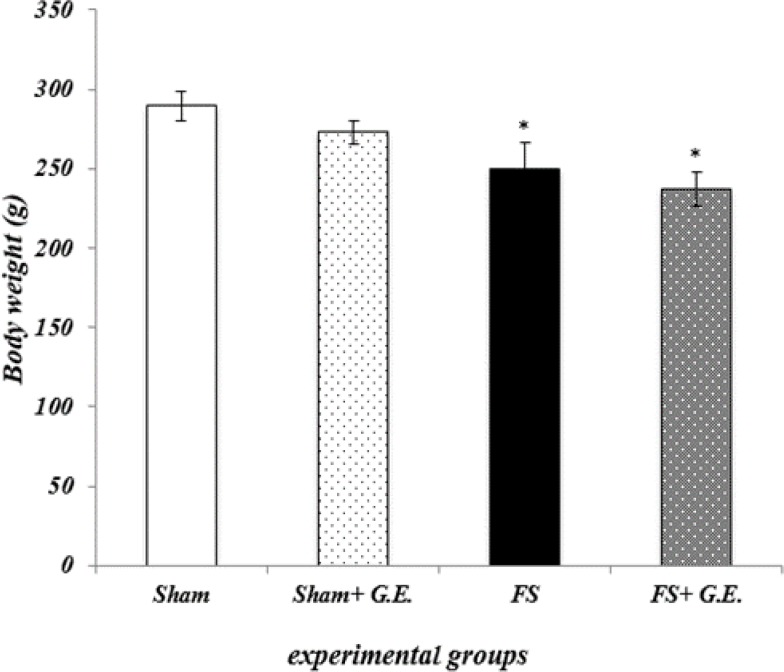
Ginger extract and ferrous sulfate-induced changes in body weight. Data are expressed as mean ± SEM (n = 7 in each group). *p<0.05 compared to the Sham and Sham + G.E groups (one-way ANOVA followed by Duncan's test

Figure 1 shows that body weights in FS group were statistically lower than Sham and Sham + G.E groups (p<0.05). FS + G.E group showed no significant changes in body weight value in comparison with FS group.

**Ginger extract and ferrous sulfate-induced changes in hepatic functional markers**

Table 1 depicts the levels of serum hepatic markers in Sham and experimental rats. In Fe-treated rats, the activities of serum liver-specific enzymes such as AST, ALT, ALP and LDH were significantly increased (p<0.01, p<0.05, p<0.001, p<0.001, respectively). Administration of ginger extract significantly reversed these changes (p<0.01, p<0.05, p<0.001, and p<0.001, for AST, ALT, ALP and LDH respectively).

Table 1 shows that serum levels of total protein, albumin, glucose, and triglyceride (p<0.001 for all cases) as well as cholesterol (p<0.01) in FS group were statistically lower than the levels of Sham and Sham + G.E groups. It also shows that serum level of total bilirubin in FS group was statistically higher than those of Sham and Sham + G.E groups (p<0.01 for both cases). Ginger extract treatment significantly reversed these changes (p<0.001 for all cases except for cholesterol (p<0.01)).

Table 2 shows that AST, ALT, ALP and LDH levels of liver tissue in FS group were statistically higher than those of Sham and Sham + G.E groups (p<0.001, p<0.001, p<0.001 and p<0.05, respectively). Ginger extract treatment reduced the levels of AST, ALT, ALP and LDH in FS + G.E in comparison to FS group (p<0.001, p<0.001, p<0.001, and p<0.05, respectively).

**Table 1:** Effect of ginger extract on iron-induced activities of serum hepatic markers in Sham and experimental rats.

Groups	Sham	Sham + GE	FS	FS + GE
AST (U/L)	82.85±5.67	74.57±6.08	439.57±118.74**††	144.28±17.38♯♯
ALT (U/L)	47.28±4.9	49.57±6.68	307.14±122.5*†	74.28±8.43♯
ALP (U/L)	31.14±3.21	31.14±4.59	189.14±19.72***†††	80.71±15.63***†††♯♯♯
LDH (U/L)	183.28±18.73	160.42±17.98	504.00±75.45***†††	71.28±35.41***††† ♯♯♯
Total bilirubin (U/L)	0.55±0.1	0.55±0.12	3.15±0.37***†††	2.25±0.28***†††♯♯♯
Total protein (mg/dl)	6.78±0.17	6.62±0.29	4.34±0.19***†††	5.82±0.23***†††♯♯♯
Albumin (mg/dl)	4.04±0.21	3.94±0.18	2.62±0.14***†††	3.03±0.19***†††♯♯♯
Triglyceride (mg/dl)	83.42±5.35	70.71±8.62	34.00±5.04***†††	57.28±6.64***♯♯♯
Cholesterol (mg/dl)	48.00±2.24	46.42±3.2	32.42±1.87**††	45.00±3.7♯♯
Glucose (mg/dl)	137.00±9.81	123.42±7.25	64.57±5.63***†††	118.14±12.8♯♯♯

* p<0.05,

** p<0.01, and

*** p<0.001 as compared to the Sham group;

† p<0.05,

†† p<0.01, and

††† p<0.001 as compared to the Sham + GE group;

♯ p<0.05,

♯♯ p<0.01, and

♯♯♯ p<0.00 as compared to the FS group.

**Table 2:** Effect of ginger extract on iron-induced activities of hepatic markers in liver tissue of Sham and experimental rats

Groups	Sham	Sham + GE	FS	FS + GE
AST (U/L)	752.85 ± 56.79	713.42± 49.88	1852.71 ± 24.58***†††	1005± 128.37**†††♯♯♯
ALT (U/L)	668.57± 32.65	684.14 ± 23.05	1719.85 ± 122.43***†††	920.14±47.24***††† ♯♯♯
ALP (U/L)	724.85 ± 40.71	639.28 ± 51.15	1412.28 ± 68.19***†††	790.71±68.91***††† ♯♯♯
LDH (U/L)	678.00± 46.99	1761.71 ± 103.79	3072.00 ± 348.7*†	2557.85 ± 199.81*†
MDA(mol/g tissue)	0.38±0.04	0.58±0.10	1.45±0.25***†††	0.46±0.10♯♯♯

* p<0.05,

** p<0.01, and

*** p<0.001 as compared to the Sham group;

† p<0.05,

†† p<0.01, and

††† p<0.001 as compared to the Sham + GE group;

♯ p<0.05,

♯♯ p<0.01, and

♯♯♯ p<0.00 as compared to the FS group.

**Table 3:** Effect of ginger extract on the levels of renal functional markers in Sham and experimental rats

Groups	Sham	Sham + GE	FS	FS + GE
Creatinine (mg/dl)	0.48± 0.03	0.47± 0.02	0.62 ± 0.01**††	0.54 ± 0.02♯♯
Urea nitrogen (mg/dl)	24.42± 2.23	25.28 ± 2.21	39.42 ± 2.70***†††	29.57 ± 1.92♯♯♯
Creatinine clearance (ml/min)	0.67±0.04	0.63±0.09	0.39±0.02*†	0.57±0.05♯
FENa (%)	0.98±0.08	1.04±0.07	1.46±0.13**††	1.01±0.07♯♯
MDA (mol/g tissue)	0.35 ± 0.04	0.42 ± 0.05	0.84 ± 0.08***†††	0.39 ± 0.05♯♯♯

* p<0.05,

** p<0.01,

*** p<0.001 as compared to the Sham group;

† p<0.05,

†† p<0.01,

††† p<0.001 as compared to the Sham + GE group;

♯ p<0.05,

♯♯ p<0.01,

♯♯♯ p<0.00 as compared to the FS group.

**Ginger extract and ferrous sulfate-induced changes in renal functional markers**

Table 3 presents the levels of renal functional markers in Sham and experimental rats. In Fe-treated rats, renal functional markers such as creatinine, urea nitrogen and fractional Na^+^-excretion (FE_Na_), were significantly increased (p<0.01, p<0.001, p<0.01, respectively), while creatinine clearance was significantly decreased (P<0.05). Administration of ginger extract significantly reversed these changes (p<0.01, p<0.001, p<0.01, and p<0.05 for creatinine, urea nitrogen and fractional Na^+^-excretion and creatinine clearance, respectively).

**Figure 2:**
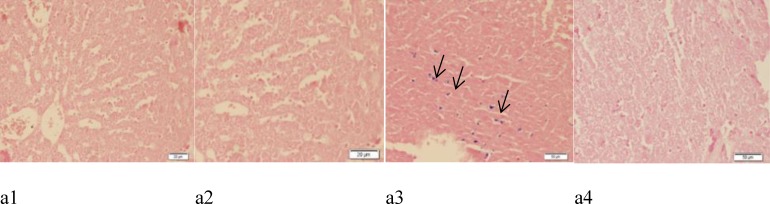
Representative light microphotographs of the livers obtained from Sham (a_1_)_, _ Sham + G.E (a2), FS (a3), and FS+G.E group (a4). (Prussian blue staining in a1, a2, a3 shows iron deposition

**Figure 3.**
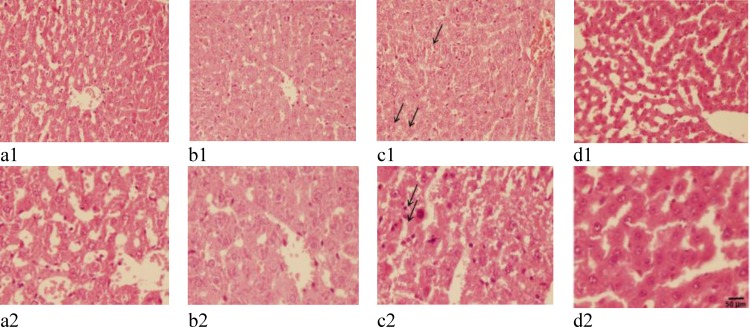
Representative light microphotographs of the livers obtained from Sham (a_1_ and a_2_)_, _ Sham + G.E (b1 and b2), FS (c1 and c2), and FS + G.E group (d1 and d2) (Hematoxylin–eosin staining in a1, b1, c1, and d1 shows swelling of hepatocyte and in a2, b2, c2, and d2 shows apoptosis

**Figure 4:**
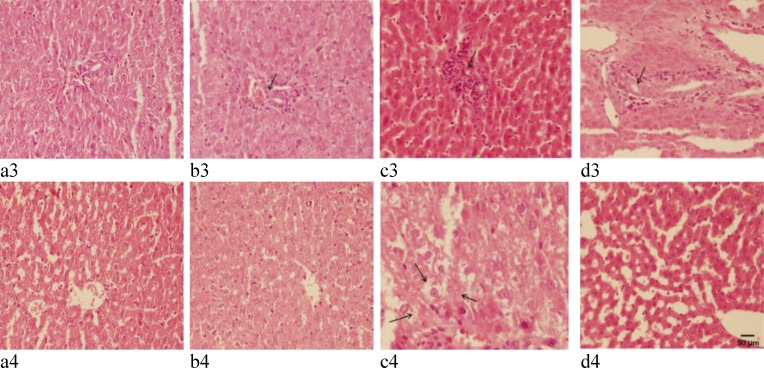
Representative light microphotographs of the livers obtained from Sham (a_1_ and a_2_)_,_ Sham + G.E (b1 and b2) and FS (c1 and c2), and FS + G.E group (d1 and d2) (Hematoxylin–eosin staining in a3, b3, c3, and d3 shows infiltration of inflammatory cells and in a4, b4, c4, and d4 shows vacuolization of cytoplasm

**Figure 5:**
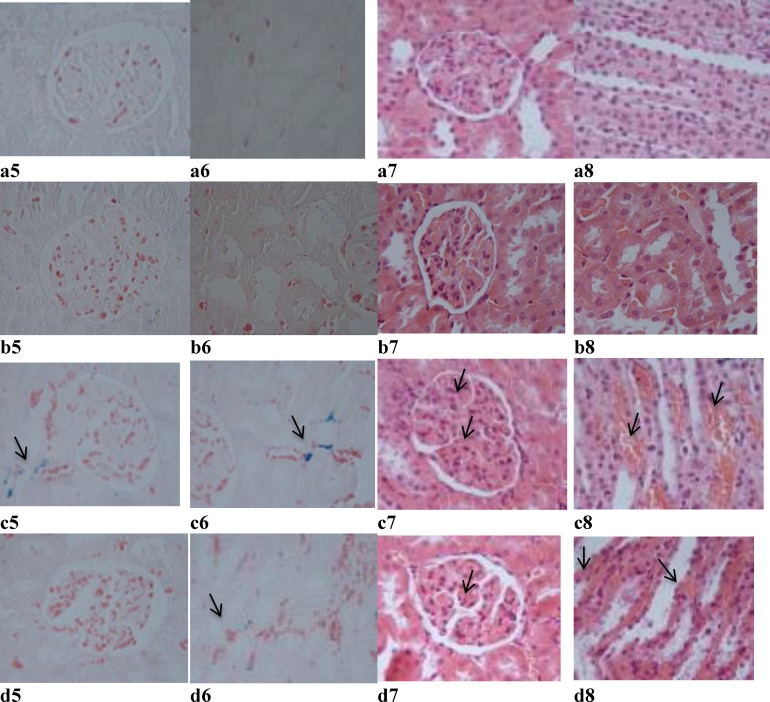
Representative light microphotographs of the kidneys obtained from Sham (a5_,_ a6, a7, and a8)_, _ Sham + G.E (b5, b6, b7, and b8), FS (c5_,_ c6, c7, and c8), and FS + G.E group (d5_,_ d6, d7, and d8). (Prussian blue staining in a5, b5, c5 and d5 shows iron particles in cytoplasm of cortical tubular cells and in a6, b6, c6 and d6 shows iron particles in cytoplasm of medullary tubular cells. Hematoxylin–eosin staining in a7, b7, c7, and d7 shows glomerular capillary congestion and in a8, b8, c8, and d8 shows medullary vascular congestion

**Ginger extract and ferrous sulfate-induced oxidative stress in the liver and kidney**

Tables 2 and 3 show that ferrous sulfate treatment resulted in significant increases in MDA contents of the liver (p<0.001) and kidney (p<0.001) compared to the Sham and Sham + G.E groups.

**Ginger extract and ferrous sulfate-induced histological damages in the liver and kidney**

Results of the histological studies of the liver were in agreement with the measured activities of hepatic enzymes. There were no abnormalities or histological changes in the livers of Sham and Sham + G.E groups (Figures 2A1, 3A1, 3A2, 3B1, 3B2, 4A3, 4A4, 4B3 and 4B4). In the FS group (Figure 2A2), iron deposition (grade 2) was observed. In FS+G.E group (Figure 2A3) less iron deposition (grade 1) was noticed in comparison with FS group. In the FS group (Figures 3C1, 3C2, 4C3, and 4C4), the most prominent lesions were hepatocellular ballooning (grade 2), apoptosis of the hepatic cells (grade 2), infiltration of inflammatory cells in the portal space (grade 3) and vacuolization of the hepatocyte cytoplasm (grade 2). In the FS + G.E group, less severe lesions were noticed in comparison with FS group (Figures 3D1, 3D2, 4D3, and 4D4). 

Renal sections from the Sham and Sham + G.E groups (Figures 5A5, 5A6, 5A7, and 5A8) exhibited minimal or no changes. In the FS group, iron deposition (grade 2) was observed in the cortex (Figure 5C5) and medulla (Figure 5C6). However, in FS + GE group (Figures 5D5 and 5D6), less iron deposition (grade 1) was noticed in comparison with FS group. Furthermore, in the FS group, there was congestion (grade 2) in glomerular capillaries (Figure 5C_7_) and medullary vessels (Figure 5C8). However, in FS + GE group (Figures 5D7 and 5D8), less congestion (grade 1) was observed in comparison with FS group. The sum of histopathological grades, marking the changes described above, is mentioned in Table 4.

**Table 4:** Total histopathological score in Sham, Sham + G.E, FS and FS + GE groups (each n=7) at the end of the experiment

Experimental groups	Liver	Kidney
Sham	0.0 ± 0.00	0.0 ± 0.00
Sham + GE	0.0 ± 0.00	0.0 ± 0.00
FS	10.87 ± 0.66^***^	7.00 ± 0.32^***^
FS + GE	4.00± 0.37^†††^	3.12± 0.29^†††^

Values are expressed as mean ± SEM.

*** p<0.001 as compared to Sham and Sham

+ GE

††† p<0.001 as compared to FS.

## Discussion

The objective of the current study was to investigate the possible protective effects of hydroalcoholic extract of ginger on ferrous sulfate (iron)-induced functional disorders and histological damages, in rats. The results showed that ginger extract significantly attenuated ferrous sulfate-induced hepatic and renal functional injuries and MDA levels in hepatic and renal tissues compared to the FS group. These results were confirmed by the histological evaluations, demonstrating significant decreases in the hepatocellular ballooning, apoptosis of the hepatic cells, infiltration of inflammatory cells in the portal space, vacuolization of the hepatocyte cytoplasm, and renal vascular congestion in the FS + G.E compared to FS group. Furthermore, ginger extract diminished iron deposition following administration of ferrous sulfate. Altogether, these results indicated that the protective effects of ginger extract may be a consequence of reduction of oxidative stress damage and iron chelating in hepatic and renal tissues.

Earlier investigations have suggested that chronic deposition of excess iron in hepatic parenchymal cells can lead to hepatic injury (Powell et al. 1980 ▶). Liver function tests can provide information on a range of disease processes (Hall and Cash 2012 ▶). In the FS group, higher enzymatic activities of tissue and serum AST and ALT (Eton and Qian 2002 ▶; Pawar et al. 2012 ▶; Selvi 2012 ▶), alkaline phosphatase (Ramaiah 2007) and LDH (Garcia-Nino and Pedraza-Chaverri 2014) in the liver tissue and serum are indicative of hepatic cellular damage because of losing functional integrity of cell membranes. In addition, bilirubin level is markedly increased in ferrous sulfate-treated rats (Pawar et al. 2012 ▶; Selvi 2012 ▶). The combination of these factors is likely to be responsible for reduced liver function following ferrous sulfate treatment in FS group. Treatment with ginger extract (400 mg/kg/day for 14 days) reduced hepatocyte damages, thereby attenuates the enzymes released into the blood. Interestingly, the membrane protective effect of ginger extract has been reported (Poorrostami et al. 2014 ▶). 

The liver has multiple important functions such as glycogen storage, detoxification, protein synthesis and hormone production. Based on our results, there was significant decreases in total protein and albumin levels in the FS group compared to Sham group. In agreement with our results, it has been shown that total protein level is decreased in ferrous sulfate-induced hepatotoxicity (Sarkar et al. 2013 ▶; Selvi 2012 ▶). Ginger extract increased total protein and albumin levels, demonstrating that ginger could ameliorate liver functions. 

In hepatocytes, Fe catalyzes the production of hydroxyl radical (OH^•^) (Hampton et al. 1998 ▶) and NO^2+^, both accelerating lipid peroxidation (Videla et al. 2003 ▶). Extreme iron deposition in the liver in the present study, was associated with the beginning and propagation of oxidative damage to biomacromolecules (Abdel-Baky et al. 2009; Aust et al. 1985; Valko et al. 2005). In the present work, ferrous sulfate treatment resulted in decreased serum glucose concentration. The liver releases glucose via by glycogenolysis and gluconeogenesis (Shrayyef and Gerich 2010). Rhodes and DePalma (1980) ▶ have suggested that imperfect energy-linked function of mitochondria impairs gluconeogenesis. Depletion of hepatic glycogen stores following uncoupling of oxidative phosphorylation, leads to hypoglycemia (Rhodes and DePalma 1980 ▶). Oral administration of ginger extract significantly inversed ferrous sulfate-induced peroxidative damage in the liver as evidenced by lower levels of MDA which might be due to the antioxidative effect of ginger extract (Jagetia et al. 2003 ▶). Besides, ginger extract increased glucose concentration to the normal levels due to its ability to reduce lipid peroxidation. Protecting ability of ginger against Fe^2+^-induced lipid peroxidation has been also shown (Ali et al. 2008 ▶; Oboh et al. 2012 ▶). Light microscopy analysis of experimental groups showed that accumulation of iron in the liver was effectively reduced by hydroalcoholic extract of ginger, which revealed that ginger extract chelates the iron (Oboh et al. 2012 ▶). It is well noticed that ginger possesses antioxidant properties (Ali et al. 2008 ▶; Oboh et al. 2012 ▶) based on which, ginger can scavenge the excess iron in biological systems.

Light microscopy for FS group showed hepatocyte ballooning and cytoplasmic vacuolization. High dose of ferrous sulfate led to changes in the levels of serum lipids in FS group. It may be due to accumulation of ferrous sulfate in the liver, which plays a principal role in lipid homeostasis. In the FS group, serum concentrations of cholesterol and TG were increased. Also, Fe-induced rise of cholesterol in serum may be due to changes in the gene expression of hepatic enzymes as suggested previously (Kojima et al. 2004 ▶). Ginger extract attenuated increased blood levels of these lipids in FS + G.E group. This reduction may be due to antioxidant ability of ginger that contributed to the protection of membrane lipids from free radicals. In agreement with our results, ginger extract diminished ballooning and vacuolization of hepatocytes in liver fibrosis induced by CCl_4_ (Motawi et al. 2011 ▶). 

In the present study, other remarkable pathological characteristics of ferrous sulfate-induced hepatotoxicity were apoptosis and leukocyte infiltration which might be due to ROS formation as iron induces oxidative stress (Czaja 2004 ▶). Administration of ginger extract reduced the histological alterations induced by ferrous sulfate. It can be attributed to the antioxidant properties of hydroalcoholic extract of ginger, which significantly reduced the oxidative stress leading to reduction of pathological changes and restoration of normal physiological functions. This is in agreement with the result of Sakr et al. (2007) who found that ginger extract diminished mancozeb-induced inflammatory cell infiltrations in the liver.

Histopathological observations showed that ferrous sulfate induced moderate glomerular capillary congestion and modest vascular congestion of the medullary tubules in the kidney. Furthermore, moderate iron deposition in cytoplasm of tubular cells in FS group was observed. Accumulation of iron in tissues results in cellular damage. Fe preferentially binds to the membrane and disturbs the redox state of the cells (Pari et al. 2015 ▶). Hence, prolonged accumulation of Fe in the tissues promotes oxidative state that leads to pathological changes in the kidney. Administration of ginger extract reduced renal histological alterations induced by ferrous sulfate. 

Moreover, increased serum levels of urea and creatinine (Table 3) are in agreement with the results reported by Pari et al. (2015) ▶ in male rats. It is recognized that elevated blood urea is correlated with an increase in protein catabolism in mammals (Harper et al. 1979 ▶). Similarly, the present results show that serum concentrations of total protein were decreased in the FS group (Table 1). Also, the increase in serum urea and creatinine concentrations as well as increased fractional excretion of Na^+ ^(FE_Na_) in the present experiment may be due to kidney dysfunction. In our model of ferrous sulfate-induced nephrotoxicity, ginger showed considerable beneficial effects, with almost complete restoration of GFR and FE_Na_ to normal levels. This prevention of the GFR decrease and FE_Na_ increase, was accompanied by less histological damages. Renal protective activity of ginger might partially be due to its antioxidant activity (Ajith et al. 2007 ▶; Hamed et al. 2012 ▶; Rodrigues et al. 2014 ▶). In this regard, administration of ginger extract significantly inversed ferrous sulfate-induced peroxidative damage in the kidney as evidenced by lower levels of MDA.

 The present study was an effort to investigate the effect of the hydroalcoholic extract of *Z.* *officinale* on hepato- and nephrotoxicity induced by ferrous sulfate in rats. This study revealed the functional disorders and histopathological damages of iron overload in the liver and kidney . Ginger extract treatment produced significant fall in serum hepatic markers, bilirubin, creatinine, BUN and tissue lipid peroxidation marker. 

In conclusion, our results indicate that ginger hydroalcoholic extract has the ability to improve liver functional biomarkers, ameliorate hepatic marker enzymes, reduce iron deposition, diminish renal functional disorders, reduce renal histological damages, and normalize the hepatic cells architecture. The hepato- and renoprotective effects of *Z. officinale* could be due to its antioxidant potential by scavenging free radicals and chelating iron. The present study therefore, provides biological evidence regarding the efficacy of ginger extract against Fe-induced toxicity in rats.
